# Incidence and Types of Human Papillomavirus Infections in Adolescent Girls and Young Women Immunized With the Human Papillomavirus Vaccine

**DOI:** 10.1001/jamanetworkopen.2021.21893

**Published:** 2021-08-23

**Authors:** Nicolas F. Schlecht, Angela Diaz, Anne Nucci-Sack, Kathleen Shyhalla, Viswanathan Shankar, Mary Guillot, Dominic Hollman, Howard D. Strickler, Robert D. Burk

**Affiliations:** 1 Department of Cancer Prevention & Control, Roswell Park Comprehensive Cancer Center, Buffalo, New York; 2 Department of Epidemiology & Population Health, Albert Einstein College of Medicine, Bronx, New York; 3 Department of Pediatrics, Mount Sinai Adolescent Health Center, Icahn School of Medicine at Mount Sinai, Manhattan, New York; 4 Departments of Pediatrics (Genetics), Microbiology & Immunology and Obstetrics, Gynecology & Women’s Health, Albert Einstein College of Medicine, Bronx, New York

## Abstract

**Importance:**

Rates of human papillomavirus (HPV) infection have decreased since the introduction of HPV vaccines in populations with high vaccine uptake. Data are limited for adolescent and young adult populations in US metropolitan centers.

**Objective:**

To determine HPV infection rates in adolescent girls and young women aged 13 to 21 years in New York City following HPV vaccination.

**Design, Setting, and Participants:**

This cohort study of type-specific cervical HPV detection was conducted at a large adolescent-specific integrated health center in New York City between October 2007 and September 2019. Participants included an open cohort of adolescent girls and young adult women who received the HPV vaccine (Gardasil; Merck & Co) over a 12-year period following HPV vaccination introduction. Data analysis was concluded September 2019.

**Exposures:**

Calendar date and time since receipt of first vaccine dose.

**Main Outcomes and Measures:**

Temporal associations in age-adjusted postvaccine HPV rates.

**Results:**

A total of 1453 participants, with a mean (SD) age at baseline of 18.2 (1.4) years, were included in the cohort (African American with no Hispanic ethnicity, 515 [35.4%] participants; African American with Hispanic ethnicity, 218 [15.0%] participants; Hispanic with no reported race, 637 [43.8%] participants). Approximately half (694 [47.8%] participants) were vaccinated prior to coitarche. Age-adjusted detection rates for quadrivalent vaccine types (HPV-6, HPV-11, HPV-16, and HPV-18) and related types (HPV-31, and HPV-45) decreased year over year, with the largest effect sizes observed among individuals who had been vaccinated before coitarche (adjusted odds ratio [aOR], 0.81; 95% CI, 0.67-0.98). By contrast, detection was higher year over year for nonvaccine high-risk cervical HPV types (aOR, 1.08; 95% CI, 1.04-1.13) and anal HPV types (aOR, 1.11; 95% CI, 1.05-1.17). The largest effect sizes were observed with nonvaccine types HPV-56 and HPV-68.

**Conclusions and Relevance:**

Whereas lower detection rates of vaccine-related HPV types were observed since introduction of vaccines in female youth in New York City, rates of some nonvaccine high-risk HPV types were higher. Continued monitoring of high-risk HPV prevalence is warranted.

## Key Points

**Question:**

Has the introduction of the vaccine for human papillomavirus (HPV) been associated with changes in infection rates among sexually active adolescent girls and young adult women?

**Findings:**

This cohort study assessed HPV infection rates of vaccinated adolescent and young adult women at a large adolescent-specific health center in New York City. Age-adjusted cervical HPV detection of vaccine-related types were lower year over year, while the prevalence of nonvaccine high-risk HPV types remained flat or higher.

**Meaning:**

These results indicate the real-world effectiveness of the HPV vaccines in female youth in New York City; nevertheless, rates of some nonvaccine high-risk HPV types were higher.

## Teaser

This cohort study of a single health center in New York City examines changes in human papillomavirus (HPV) infection rates among adolescent girls and young women following the introduction of a quadrivalent vaccine.

## Introduction

Human papillomavirus (HPV) infections remain the most common sexually transmitted infection in female adolescents and young adults, and are responsible for the development of anogenital warts, cervix cancer, and the majority of anal and oropharyngeal cancers.^1,2^ The quadrivalent vaccine has been shown to be highly effective at reducing the incidence of cervical and anogenital HPV infections and related diseases caused by vaccine types (HPV-6, HPV-11, HPV-16, and HPV-18),^3,4,5,6^ along with 2 phylogenetically related high-risk HPV types (HPV-31 and HPV-45) that benefitted from cross-protection by the quadrivalent vaccine.^7,8,9,10,11,12^ With the introduction of the 9-valent HPV vaccine in 2017, protection was extended to 5 additional high-risk HPV types (HPV-31, HPV-45, HPV-33, HPV-52, and HPV-58).

Based on the prevaccine era population estimates,^13^ eliminating the above 9 HPV types would prevent over 90% of cervical and anal neoplasia^8,14,15,16^ and anogenital warts.^17^ Whereas vaccination rates in countries where national programs were introduced early have been higher,^7,10,18,19,20,21,22^ uptake of the HPV vaccine in the US has been slow.^23,24,25,26^ Furthermore, the current vaccines do not provide immunity to all HPV types, including some nonvaccine types that can lead to cancer.^27^ While nonvaccine high-risk HPV types (ie, HPV-35, HPV-39, HPV-51, HPV-56, HPV-59, and HPV-68) collectively account for less than 10% of all cervical and anal cancers in women,^28^ 1 or more of these types are detected in a majority (56.1%) of sexually active 20- to 24-year-old US women,^12^ and the prevalence of some nonvaccine types (like HPV-35 and HPV-59) have been found to be higher among racial/ethnic minority groups.^14,29,30,31^

However, there is a paucity of longitudinal HPV-vaccine effectiveness data in adolescent and young adult populations in major US cities. This open cohort study (conducted between 2007 and 2019) assessed type-specific high-risk HPV detection rates in cervical and anal specimens of sexually active girls and women aged 13 to 21 years at enrollment in a longitudinal study conducted at a large adolescent-specific health center that provides integrated health services in New York City. Temporal associations in age-adjusted postvaccine HPV rates were evaluated in a large population of adolescent girls and young adult women in a metropolitan setting.

## Methods

### Study Population

This was a longitudinal evaluation in an open cohort of adolescent girls and young adult women enrolled prospectively over a period of approximately 12 years.^32,33^ Shortly after the approval of the first HPV vaccine in 2007, we initiated a prospective cohort study of sexually active female adolescents and young adults at the Mount Sinai Adolescent Health Center. Patients aged 13 to 21 years attending the clinic were invited to participate in a prospective study with repeated assessments every 6 months until age 26 years. Girls and women were eligible to participate if they had already engaged in sexual intercourse and intended to receive or had already received the Gardasil vaccine (Merck & Co). Girls and women who were pregnant at the time of recruitment were invited to return after their pregnancy. Written informed consent was collected from all participants prior to enrollment. The study was approved by the institutional review board at the Icahn School of Medicine at Mount Sinai. This study followed the Strengthening the Reporting of Observational Studies in Epidemiology (STROBE) reporting guideline.

### Study Visits and Specimens

Study participants received a gynecological examination and completed a health history interview at each visit, including assessment of sexual behaviors, history of sexually transmitted infection, and immunizations. In addition, a self-report questionnaire was completed at each visit to assess lifetime and recent sexual activity and condom use. Vaccination status was confirmed using the New York City and state immunization registries.

Specimen collection was performed at each 6-month visit by clinicians following previously described protocols.^32,33^ Briefly, cervical cells were obtained using a cytobrush placed in PreservCyt medium (Hologic) following the same procedure as for Pap smears. Anal cells were also collected in PreservCyt using a polyethylene terephthalate swab moistened in tap water. Specimens were stored at −20 °C immediately following collection.

### DNA Extraction and HPV Typing

HPV infection outcomes were based on DNA of cervical and anal samples using a well-established polymerase chain reaction (PCR)–based protocol.^34,35^ Briefly, samples were processed in a biosafety cabinet in a laboratory that was physically separate from where the PCR amplifications were performed to reduce the risk of contamination. DNA was purified from samples using DNA mini spin columns (Qiagen) according to the manufacturer’s protocols. Five microliters of purified DNA were then amplified in a 100-μl reaction using gold Taq and PCR with a mix of MY09/MY11-L1 consensus primers and a set of type-specific primers that amplify a 450-base pair HPV DNA fragment and improve the performance of the system. PC04/GH20 primers, which simultaneously amplify a 268-base pair cellular β-globin DNA fragment, were included as an internal control.^34,35^ Ten microliters of the PCR reaction mix were analyzed by gel electrophoresis to validate successful amplification.

Samples were then analyzed for HPV DNA type by oligonucleotide allele hybridization. Seven microliter aliquots of the initial PCR reaction were applied to 10 replicate filters using a 96-well pipetting system. Filters were individually hybridized using biotinylated type-specific oligonucleotide probes for over 40 alpha HPV types known to infect the mucosal epithelia, including vaccine types and nonvaccine high-risk HPV types^27^ as well as other low-risk types associated with anogenital warts.^36^ Hybridization signals were graded using a validated scale for signal intensity.^37^

### Statistical Analysis

To assess whether rates of HPV infection have changed since the introduction of the vaccine, we estimated the age-adjusted prevalence and 95% CIs by calendar date (ie, years since 2008) and time since vaccination (calculated by subtracting participant age at study visit from age at first vaccine dose). We also assessed for possible breakthrough infections with vaccine types by restricting analyses to participants who initiated vaccination prior to coitarche (determined based on the difference between age at first vaginal intercourse and age at first dose). HPV types were grouped based on their relationship to the vaccine and oncogenic potential. Outcomes included prevalence and incidence of: (1) vaccine-related HPV types, representing the types affected by the quadrivalent vaccine (HPV-6, HPV-11, HPV-16, HPV-18, HPV-31, and HPV-45) and the more recent 9-valent vaccine (HPV-6, HPV-11, HPV-16, HPV-18, HPV-31, HPV-33, HPV-45, HPV-52, and HPV-58); (2) nonvaccine high-risk HPV types (HPV-35, HPV-39, HPV-51, HPV-56, HPV-59, and HPV-68) associated with increased risk of cancer^27^; and (3) nonvaccine low-risk types identifiable by our PCR assay. Additional analyses looking at individual HPV types were also performed.

We evaluated whether infection rates for HPV have changed since the administration of the vaccine by assessing longitudinally the probability of HPV detection over time among vaccinated participants while adjusting for changes in cohort characteristics over time. We used a multivariable logistic regression approach with generalized estimating equations for open cohorts,^38,39,40^ with time since introduction of the vaccine as the primary independent variable. As age was a metameter of time, we used age as a within-patient time-varying covariate and year of cohort entry as a time-fixed covariate.

Potential confounding factors were adjusted for in the multivariable models. Models for vaccine types were adjusted using number of vaccine doses received prior to the study visit (1 or 2 vs 3), age at coitarche (in years), age of first vaccine dose (in years), and number of recent partners in the past 6 months (less than 2, 2, or more than 3). Additional confounding factors and risk factors for cervical high-risk HPV adjusted for included consistency of condom use during recent sex (always vs less than always) and history of a positive chlamydia test, the next most common sexually transmitted infection (yes vs no, unknown), which was updated at each visit to account for any new infections. For anal HPV, we adjusted for whether participants reported having anal sex in the prior 6 months (yes vs no) in addition to other variables that we fitted in the cervical HPV model. To address potential masking effects caused by differences in assay sensitivity to vaccine types over nonvaccine types reported with some PCR systems,^41^ we adjusted for concurrent detection of vaccine types in the models looking at nonvaccine high-risk HPV types.

We used a last value carry forward approach to impute missing data for the variables: number of recent partners in the past 6 months and history of chlamydia. As the proportion of missing information for other covariates was negligible, they were modeled under a missing completely at random assumption. The functional forms of variables and model goodness of fits were assessed using cumulative residuals. We assessed the correlations between model variables using variance inflation factors and empirical correlation coefficients, and centered age at 13 years (the minimum age of entry into the cohort) to reduce collinearity. The within-patient correlations over repeated measures were modeled using exchangeable structure based on quasi-likelihood information criterion, and robust standard errors were used for inference. All analyses were performed using SAS version 9.4 (SAS Institute). The threshold for significance was *P* < .05 in 2-sided tests.

## Results

The study cohort included 1506 adolescent girls and young women enrolled between October 2007 and September 2019, with a mean (SD) age at baseline of 18.2 (1.4) years. Accrual occurred throughout the period of study with approximately half of the cohort enrolled before May 2012. Thirty-five participants did not complete a postvaccination visit, and HPV typing results were not available from any visit for 18 participants, leaving a final analytical cohort of 1453 vaccinated adolescent females (eFigure 1 in the Supplement). Participants were observed for a median (interquartile range [IQR]) of 2.4 years (7.4 months to 5.3 years). Over half of participants (855 [58.8%]) identified as Hispanic and approximately half (733 [50.4%]) as African American, including 218 (15.0%) who identified as both African American and Hispanic (Table).

**Table.** Baseline Characteristics of Cohort at Study Entry

Characteristic	Full vaccinated cohort (n = 1453)			Cohort vaccinated before coitarche (n = 694)		
	Participants, No. (%)	Cervical HPV positive at baseline, No. (%)	Anal HPV positive at baseline, No. (%)	Participants, No. (%)	Cervical HPV positive at baseline, No. (%)	Anal HPV positive at baseline, No. (%)
Age at first postvaccine visit, y						
13-14	27 (1.9)	14 (51.9)	9 (33.3)	19 (2.7)	11 (57.9)	7 (36.8)
15-16	255 (17.5)	112 (43.9)	83 (32.5)	156 (22.5)	62 (39.7)	48 (30.8)
17-18	689 (47.4)	332 (48.2)	224 (32.5)	347 (50.0)	149 (42.9)	111 (32.0)
19-21	482 (33.2)	221 (45.9)	163 (33.8)	172 (24.8)	73 (42.4)	53 (30.8)
Race/ethnicity						
African American/Hispanic	218 (15.0)	110 (50.5)	74 (33.9)	117 (16.9)	53 (45.3)	40 (34.2)
African American/not Hispanic	515 (35.4)	269 (52.2)	187 (36.3)	213 (30.7)	105 (49.3)	72 (33.8)
No reported race/Hispanic	637 (43.8)	261 (41.0)	197 (30.9)	333 (48.0)	126 (37.8)	104 (31.2)
Other race/not Hispanic^a^	63 (4.3)	24 (38.1)	12 (19.0)	27 (3.9)	9 (33.3)	2 (7.4)
Unknown race	20 (1.4)	15 (75.0)	9 (45.0)	4 (0.6)	2 (50.0)	1 (25.0)
Year entered in study						
2007-2010	430 (29.6)	195 (45.3)	144 (33.5)	75 (10.8)	30 (40.0)	24 (32.0)
2011-2013	513 (35.3)	224 (43.7)	148 (28.8)	250 (36.0)	88 (35.2)	66 (26.4)
2014-2016	242 (16.7)	114 (47.1)	72 (29.8)	166 (23.9)	72 (43.4)	47 (28.3)
2017-2019	268 (18.4)	146 (54.5)	115 (42.9)	203 (29.3)	105 (51.7)	82 (40.4)
Lifetime No. of sex partners at inclusion						
1	265 (18.2)	75 (28.3)	55 (20.8)	171 (24.6)	49 (28.7)	35 (20.5)
2	248 (17.1)	99 (39.9)	62 (25.0)	143 (20.6)	50 (35.0)	38 (26.6)
3-4	430 (29.6)	234 (54.4)	161 (37.4)	200 (28.8)	104 (52.0)	74 (37.0)
≥5	501 (34.5)	267 (53.3)	198 (39.5)	179 (25.8)	92 (51.4)	72 (40.2)
Unknown	9 (0.6)	4 (44.4)	3 (33.3)	1 (0.1)	0	0
No. of sex partners in past 6 mo						
0	52 (3.6)	14 (26.9)	13 (25.0)	30 (4.3)	5 (16.7)	6 (20.0)
1	813 (56.0)	332 (40.8)	231 (28.4)	394 (56.8)	142 (36.0)	110 (27.9)
2	339 (23.3)	185 (54.6)	133 (39.2)	167 (24.1)	87 (52.1)	56 (33.5)
≥3	236 (16.2)	143 (60.6)	98 (41.5)	103 (14.8)	61 (59.2)	47 (45.6)
Unknown	13 (0.9)	5 (38.5)	4 (30.8)	0	0	0
Frequency of condom use						
Never or sometimes	1161 (79.9)	570 (49.1)	401 (34.5)	548 (79.0)	255 (46.5)	193 (35.2)
Always	288 (19.8)	109 (37.8)	76 (26.4)	146 (21.0)	40 (27.4)	26 (17.8)
Unknown	4 (0.3)	0	2 (50.0)	0	0	0
Anal sex in past 6 mos						
No	1229 (84.6)	557 (45.3)	396 (32.2)	586 (84.4)	229 (39.1)	172 (29.4)
Yes	224 (15.4)	122 (54.5)	83 (37.1)	108 (15.6)	66 (61.1)	47 (43.5)
Ever tested positive for chlamydia						
No	770 (53.0)	323 (41.9)	207 (26.9)	358 (51.6)	130 (36.3)	95 (26.5)
Yes	484 (33.3)	276 (57.0)	210 (43.4)	192 (27.7)	113 (58.9)	85 (44.3)
Unknown	199 (13.7)	80 (40.2)	62 (31.2)	144 (20.7)	52 (36.1)	39 (27.1)
Years since first intercourse						
<1	107 (7.4)	36 (33.6)	30 (28.0)	94 (13.5)	31 (33.0)	25 (26.6)
1 to <2	281 (19.3)	125 (44.5)	94 (33.5)	198 (28.5)	85 (42.9)	68 (34.3)
2 to <3	347 (23.9)	177 (51.0)	110 (31.7)	171 (24.6)	82 (48.0)	53 (31.0)
3 to <4	294 (20.2)	149 (50.7)	102 (34.7)	141 (20.3)	63 (44.7)	44 (31.2)
≥4	411 (28.3)	186 (45.3)	140 (34.1)	90 (13.0)	34 (37.8)	29 (32.2)
Unknown	13 (0.9)	6 (46.2)	3 (23.1)	0	0	0
Doses received prior to entry into cohort						
1	180 (12.4)	92 (51.1)	65 (36.1)	25 (3.6)	8 (32.0)	7 (28.0)
2	320 (22.0)	163 (50.9)	122 (38.1)	61 (8.8)	24 (39.3)	20 (32.8)
≥3	953 (65.6)	424 (44.5)	292 (30.6)	608 (87.6)	263 (43.3)	192 (31.6)
Age at first vaccine dose, y						
<13	373 (25.7)	154 (41.3)	115 (30.8)	365 (52.6)	153 (41.9)	114 (31.2)
13-14	333 (22.9)	157 (47.1)	112 (33.6)	230 (33.1)	101 (43.9)	74 (32.2)
15-16	368 (25.3)	177 (48.1)	115 (31.3)	88 (12.7)	37 (42.0)	29 (33.0)
17-21	365 (25.1)	186 (51.0)	136 (37.3)	11 (1.6)	4 (36.4)	2 (18.2)
Unknown	14 (1.0)	5 (35.7)	1 (7.1)	0	0	0
Time since first dose to inclusion, y						
<1	509 (35.0)	264 (51.9)	190 (37.3)	40 (5.8)	18 (45.0)	13 (32.5)
2-3	446 (30.7)	204 (45.7)	134 (30.0)	200 (28.8)	87 (43.5)	63 (31.5)
4-5	148 (10.2)	57 (38.5)	40 (27.0)	257 (37.0)	100 (38.9)	75 (29.2)
≥6	336 (23.1)	149 (44.3)	114 (33.9)	197 (28.4)	90 (45.7)	68 (34.5)
Unknown	14 (1.0)	5 (35.7)	1 (7.1)	0	0	0

Abbreviation: HPV, human papillomavirus.

^a^ Included White, Asian, American Indian and Pacific Islander, and unspecified.

The proportions of new participants who identified as African American or Hispanic did not change over the course of the study. Most participants (931 [64.1%]) reported having 3 or more lifetime sexual partners at baseline. Mean (SD) age at first intercourse did not significantly change over time—from 15.2 (0.5) years in 2008 to 15.4 (0.7) in 2019 (2-sided *t* test *P* = .95), whereas age at vaccine initiation went from 18.6 (0.5) years in 2008 to 12.4 (0.5) years in 2019 (*P* < .001) (eFigure 2 in the Supplement).

A total of 694 participants received the vaccine before coitarche with a mean (SD) age at vaccine initiation of 13.0 (1.8) years and age at study entry of 17.9 (1.3) years. Participants in this subcohort were more likely to be enrolled after 2012 and were observed for a median of 2.0 years (IQR, 6.1 months to 4.4 years). Subcohort participants were also younger at time of entry, more likely to be vaccinated younger, had fewer lifetime sexual partners, were less likely to engage in anal sex, and were enrolled into the study sooner after becoming sexually active (Table).

**Figure 1.**
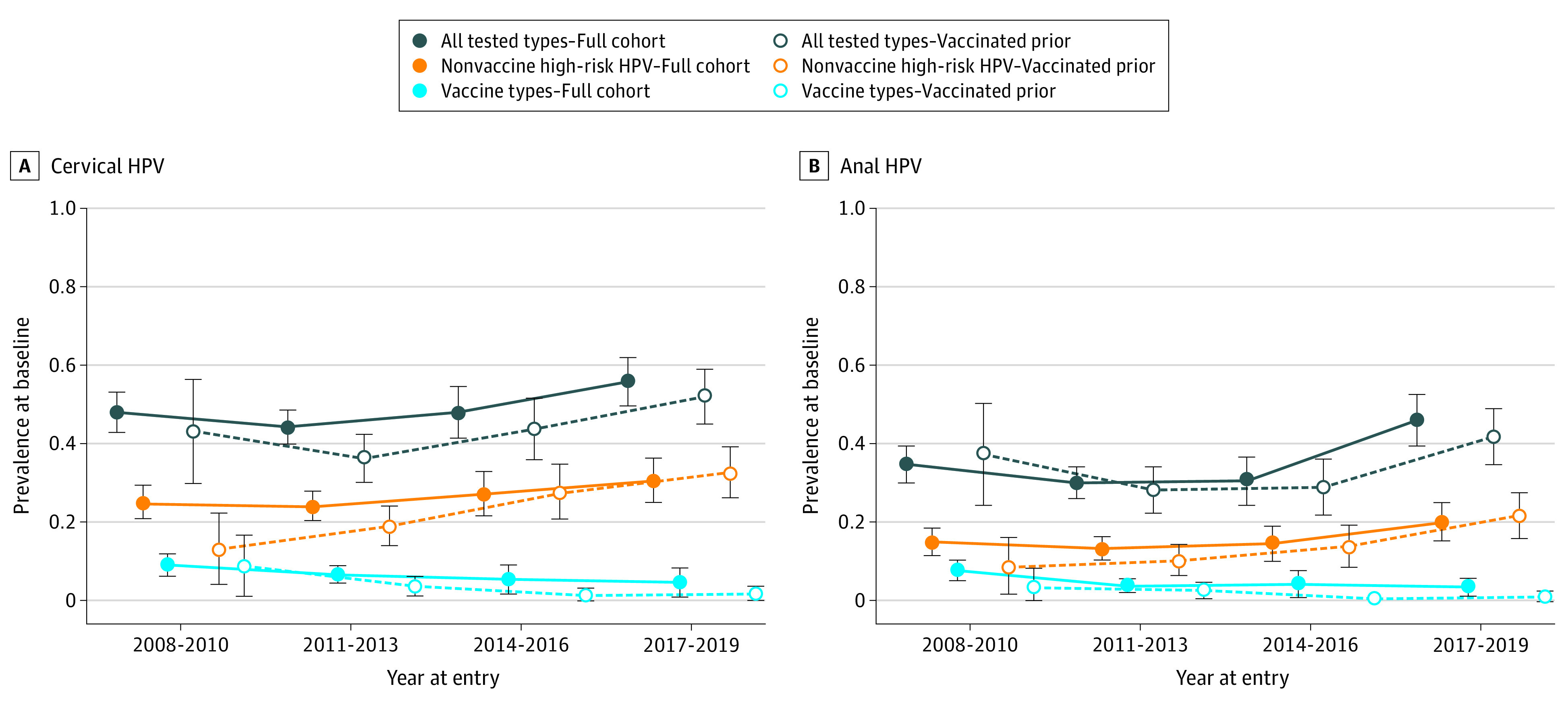
Age-Standardized Prevalence of Cervical and Anal HPV Among Vaccinated Adolescent Girls and Young Women by Year of Entry HPV indicates human papillomavirus.

Age-adjusted prevalence estimates showed a lower prevalence of cervical vaccine types over time since introduction of the quadrivalent vaccine, from 9.1% (95% CI, 6.2%-11.9%) between 2008 and 2010 to 4.7% (95% CI, 1.2%-8.2%) between 2017 and 2019 (*P* = .004) in the overall cohort, and from 8.8% (95% CI, 1.1%-16.6%) to 1.7% (95% CI, 0%-3.7%; *P* = .003) in the subcohort vaccinated prior to coitarche (Figure 1). Similar relative differences in age-adjusted prevalence of anal vaccine types were also observed. Surprisingly, in contrast to vaccine types, we observed higher prevalence of cervical and anal HPV overall, and of nonvaccine high-risk HPV types specifically. Age-adjusted prevalence of cervical nonvaccine high-risk HPV types at study entry was 25.1% (95% CI, 20.9%-29.4%) between 2008 and 2010 and 30.5% (95% CI, 24.9%-36.1%) between 2017 and 2019 (*P* = .03). Stratification on participants’ age at entry and recent number of sexual partners did not change the observed trends (eFigures 3 and 4 in the Supplement).

**Figure 2.**
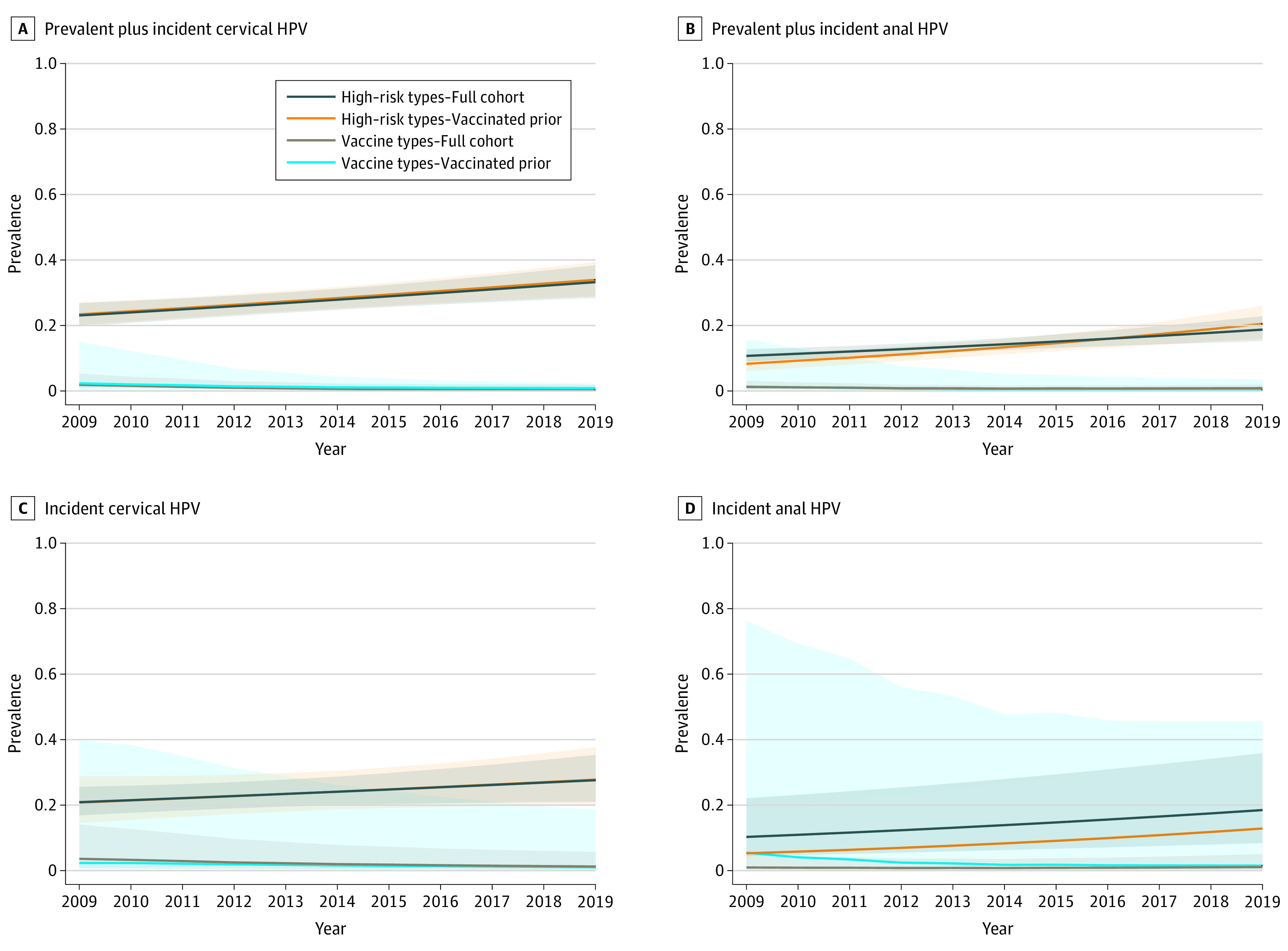
Adjusted Probability of Cervical and Anal Prevalent and Incident HPV Infections Over Time Shaded regions indicate 95% CIs; HPV, human papillomavirus. Predicted probability estimates were based on a typical vaccinated cohort participant aged 18 years who received the vaccine prior to coitarche, had 2 partners in the prior 6 months, inconsistently used condoms during recent sex (for cervical HPV), had no history of chlamydia, had no history of anal sex (for anal HPV), and were negative for a vaccine type concurrently at the same site. For vaccine types, probability estimates were also based on a participant who had received all recommended vaccine doses and who had initiated sexual activity after receipt of their first vaccine dose at the mean dose age among participants enrolled that year (eg, 15.6 years in 2009, 14.3 years in 2012, 12.5 years in 2015, 12.1 years in 2018). Estimates were derived by multivariable logistic regression with generalized estimating equations adjusting for intraparticipant correlations between repeat visit measures (assuming exchangeable correlation).

We also assessed for the likelihood of HPV infection by nonvaccine types adjusting for changes in risk factors over time (eTable in the Supplement). Among participants who were vaccinated prior to coitarche, we observed significant inverse associations (ie, protection) for cervical vaccine types over time at a rate of 19% per year (adjusted odds ratio [aOR], 0.81; 95% CI, 0.67-0.98). In contrast, significant positive associations were detected for cervical nonvaccine high-risk HPV types at a rate of 8% per year (aOR, 1.08; 95% CI, 1.07-1.13), and 11% per year for anal nonvaccine high-risk HPV (aOR, 1.11; 95% CI, 1.05-1.17). These changes were independent of concurrent detection of vaccine types. We also observed similar, but not significant, inverse and positive correlations with detection of incident cervical vaccine and nonvaccine types over time, respectively. Using the adjusted relative odds from these longitudinal models, we generated probability estimates over time assuming a typical vaccinated female participant who was vaccinated prior to coitarche (Figure 2).

**Figure 3.**
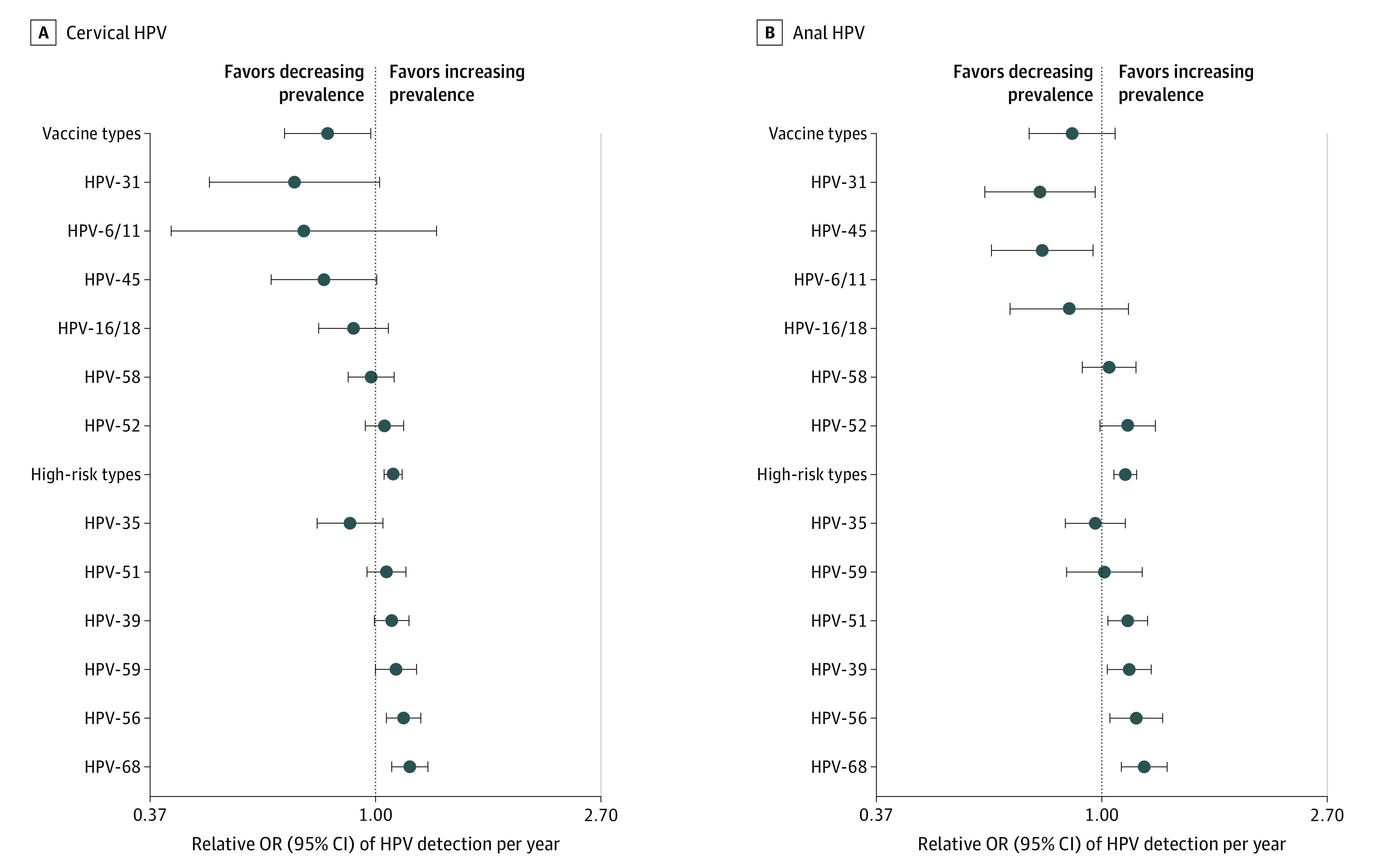
Change in Relative Odds of Detection per Year of Individual Cervical and Anal Human Papillomavirus (HPV) Vaccine Types and Nonvaccine High-Risk HPV Types Odds ratios (ORs) and 95% CIs (indicated by the error bars) were estimated by multivariable logistic regression with generalized estimating equations adjusting for intraparticipant correlations between repeat visit measures (assuming exchangeable correlation) and for covariates listed in eTable in the Supplement. An OR of 1.0 (indicated by the vertical plot line) represents no change in prevalence by year. ORs lower than 1.0 represent decreasing prevalence, and ORs greater than 1.0 represent increasing prevalence. Estimates for cervical type HPV-33 and anal types HPV-33 and HPV-6/11 are not shown due to insufficient numbers of events to generate stable models.

The changes in annual detection rates over time were more apparent for some individual HPV types (Figure 3). Significant inverse associations with time were seen for vaccine-related types HPV-31 (anal: aOR, 0.76; 95% CI, 0.60-0.97) and HPV-45 (anal: aOR, 0.77; 95% CI, 0.61-0.96). By contrast, significant positive associations with detection of nonvaccine cervical high-risk HPV types were seen for HPV-56 (aOR, 1.13; 95% CI, 1.05-1.22), HPV-59 (aOR, 1.09; 95% CI, 1.00-1.20), and HPV-68 (aOR, 1.16; 95% CI, 1.07-1.26), and for anal high-risk HPV types HPV-39 (aOR, 1.13; 95% CI, 1.02-1.24), HPV-51 (aOR, 1.12; 95% CI, 1.03-1.22), HPV-56 (aOR, 1.16; 95% CI, 1.04-1.31), and HPV-68 (aOR, 1.21; 95% CI, 1.09-1.33).

## Discussion

In a prospective open cohort study of adolescent and young adult females living in New York City, we found that age-adjusted detection rates of HPV vaccine types were associated with lower rates of HPV since the vaccine was introduced. We observed higher associated rates of type-specific inverse associations with HPV-6/11, HPV-16, HPV-18, HPV-31, and HPV-45, which are targeted by the vaccines. However, in contrast to vaccine types, we observed higher rates of anal and cervical nonvaccine types, including high-risk HPV types 39, 51, 56, and 68.

As reported in other studies,^42^ we did not find differences in the reported number of sexual partners following the introduction of the vaccine. Instead, the adolescent girls and young women in this study have been getting vaccinated earlier, consistent with recent national trends.^43^ Nonetheless, we observed a positive association with time for overall HPV prevalence in this cohort after controlling for time since coitarche and number of recent sexual partners. Whereas type replacement has been suggested as a reason for the higher prevalence of nonvaccine high-risk HPV in some populations,^44,45,46,47^ it was not seen in clinical trial cohorts.^48^ Although we observed a higher prevalence of specific nonvaccine types over time (eg, HPV-39, HPV-51, HPV-56, and HPV-68), we interpret this as insufficient to suggest type replacement at this time. Moreover, population evidence of cross-immunization protection against types genetically related to HPV-16, and to a lesser extent HPV-18, in vaccinated women has been reported by others.^46,49^

One of the unique contributions that this study provides is the evaluation of a real-world example of the HPV infection rates following immunization in a population of adolescent girls and young adult women at a single health center in a large US city, reflecting strong evidence of vaccine effectiveness. Previous surveillance studies from the US have involved older women and populations with relatively low vaccine coverage.^4,5,6,50,51,52^ Recent results, however, have found that the overall prevalence of HPV, including vaccine and nonvaccine types, has declined since the prevaccine era among vaccinated girls and women aged 14 to 19 years.^53^ By contrast, increases in nonvaccine high-risk HPV types have been reported in some countries with national school-based vaccine programs.^12,48^ These secular trends have important public health implications for prevention of cervical cancer using HPV screening in vaccinated populations. For example, HPV-68 represents a high-risk HPV type that has recently been reported to be increasing in some populations.^54^ Nevertheless, underlying mechanisms for the observed increased detection of specific types over time in a vaccinated cohort are not clear, as evidence for type replacement as a biological mechanism is lacking.

### Limitations

Some limitations of our study require consideration. First, we did not measure for changes in persistent HPV infections or cytological end points over time. Assuming HPV clearance rates of 6 to 12 months, as has been reported for cervical and anal HPV,^55,56^ it is likely that the higher detection rates observed among recent enrollees may be a result of transient infections. Longer-term follow-up and a larger sample size would be required to assess more clinically relevant outcomes. It is also important to emphasize that while the prevalence of nonvaccine HPV may be increasing in our study cohort, the absolute risk and population attributable fractions of cancer for nonvaccine types are much lower than of vaccine types, which themselves have been declining.^50,53^ Moreover, population-wide reductions in HPV-associated diseases are already being seen.^7,10,18,19,20,21,22^

Second, the elimination of HPV-16, which can produce a strong signal on L1-primer–based PCR assays, has been associated with higher detection of nonvaccine HPV types competing for the same primers, including HPV-52 and HPV-58.^41,46^ However, given the low prevalence of HPV-16 (and other quadrivalent vaccine types) in this vaccinated cohort, we anticipated that the effect of masking would be minimized. Our regression analyses indicated masking was unlikely, as we did not see significantly higher prevalence rates over time for HPV-52 or HPV-58 with lower HPV-16 prevalence among vaccinated participants.

Lastly, because being sexually active was an inclusion criterion for participation in our study, there was likely significant HPV exposure in the study population before enrollment and potentially at time of vaccination. We attempted to correct for this by showing results restricted to participants who were vaccinated before coitarche. We also adjusted for other significant indicators of HPV exposure, including recent number of sexual partners and history of chlamydia. Nonetheless, we cannot confirm if detection of vaccine types after vaccination reflected breakthrough infections, or, as noted, simply detection of a prior (or transient) HPV infection.

## Conclusions

This cohort study provides real-world evidence of lower detection rates of vaccine-targeted HPV types following immunization of female youth in New York City,^33^ with now over 10 years of data in this cohort. However, our study findings suggest that postvaccination rates of nonvaccine high-risk HPV types may be greater in some higher-risk groups.^5^ As such, surveillance with HPV testing should be considered. While we did observe increases for some nonvaccine high-risk HPV types in this cohort, the clinical implications remain to be determined as the attributable risks of cancer associated with most nonvaccine high-risk HPV types remain low.^28,31^

## Notes

Supplement. **eFigure 1.** Flowchart of Participants Included by Year of Entry and Follow-up Points **eFigure 2.** Changes in Age at Coitarche and Vaccine Dose by Year of Study Entry **eFigure 3.** Prevalence of Cervical and Anal HPV by Year and Age Category **eFigure 4.** Prevalence of Cervical HPV by Year and Number of Partners **eTable.** Adjusted Relative Odds of Detection of Cervical and Anal HPV Over Time
