# Supplementary material for: Incidence and Types of Human Papillomavirus Infections in Adolescent Girls and Young Women Immunized With the Human Papillomavirus Vaccine
Source: JAMA Netw Open. 2021 Aug 23;4(8):e2121893. doi: 10.1001/jamanetworkopen.2021.21893 (PMC8383132; doi:10.1001/jamanetworkopen.2021.21893)
Supplement: Supplement. — eFigure 1. Flowchart of Participants Included by Year of Entry and Follow-up Points eFigure 2. Changes in Age at Coitarche and Vaccine Dose by Year of Study Entry eFigure 3. Prevalence of Cervical and Anal HPV by Year and Age Category eFigure 4. Prevalence of Cervical HPV by Year and Number of Partners eTable. Adjusted Relative Odds of Detection of Cervical and Anal HPV Over Time [file jamanetwopen-e2121893-s001.pdf]

Supplemental Online Content

Schlecht NF, Diaz A, Nucci-Sack A, et al. Incidence and types of human papillomavirus infections in adolescent girls and young women immunized with the human papillomavirus vaccine. *JAMA Netw Open.* 2021;4(8):e2121893. doi:10.1001/jamanetworkopen.2021.21893

eFigure 1. Flowchart of Participants Included by Year of Entry and Follow-up Points

eFigure 2. Changes in Age at Coitarche and Vaccine Dose by Year of Study Entry

eFigure 3. Prevalence of Cervical and Anal HPV by Year and Age Category

eFigure 4. Prevalence of Cervical HPV by Year and Number of Partners

eTable. Adjusted Relative Odds of Detection of Cervical and Anal HPV Over Time

This supplemental material has been provided by the authors to give readers additional information about their work.

© 2021 Schlecht NF et al. *JAMA Network Open.*

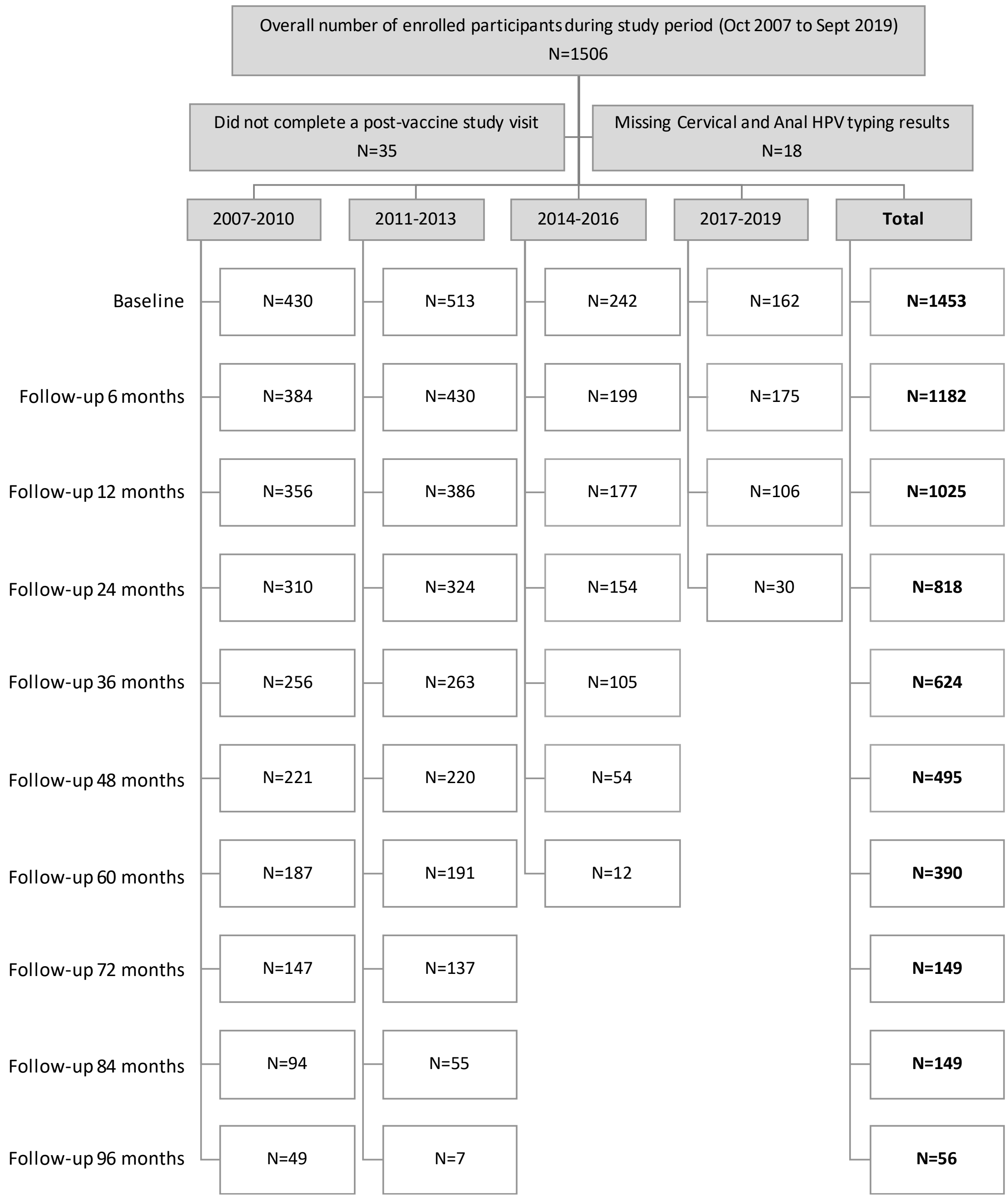

eFigure 1. Flowchart of participants included by year of entry and follow-up points

© 2021 Schlecht NF et al. *JAMA Network Open.*

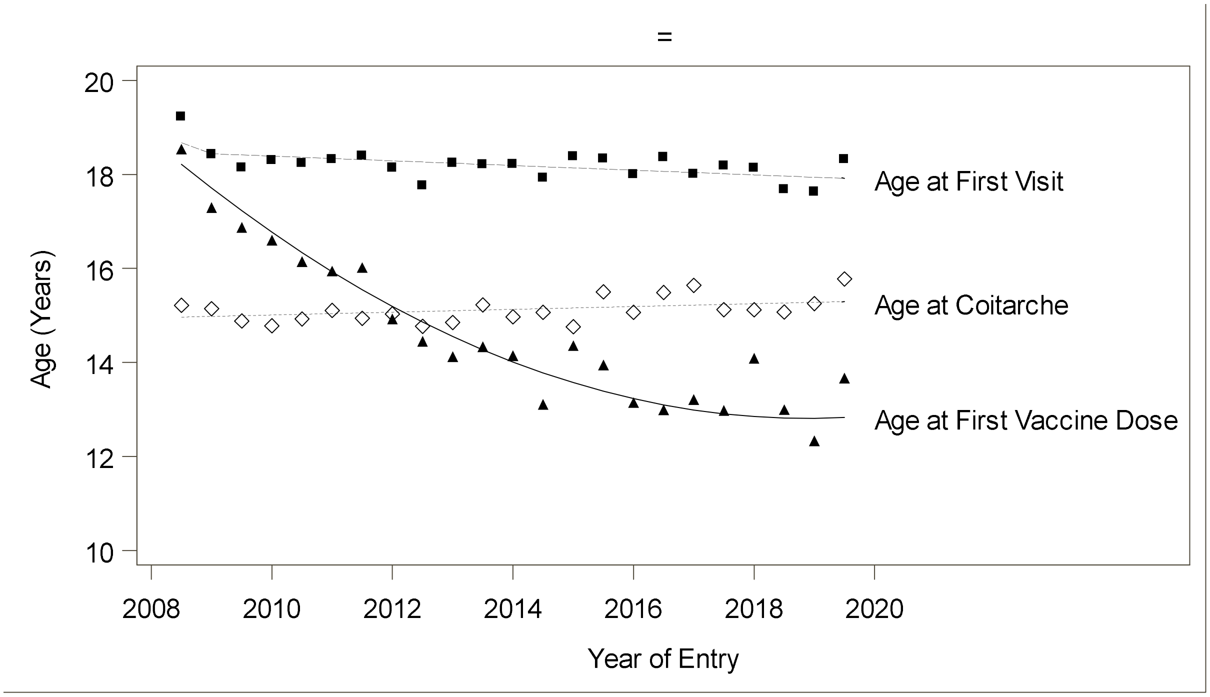

eFigure 2. Changes in age at coitarche and vaccine dose by year of study entry.

Shown are mean age at entry (filled squares), mean age at coitarche (empty diamonds), and mean age at first vaccine dose (filled triangles). Data were averaged by half year.

© 2021 Schlecht NF et al. *JAMA Network Open.*

A-Cervical HPV

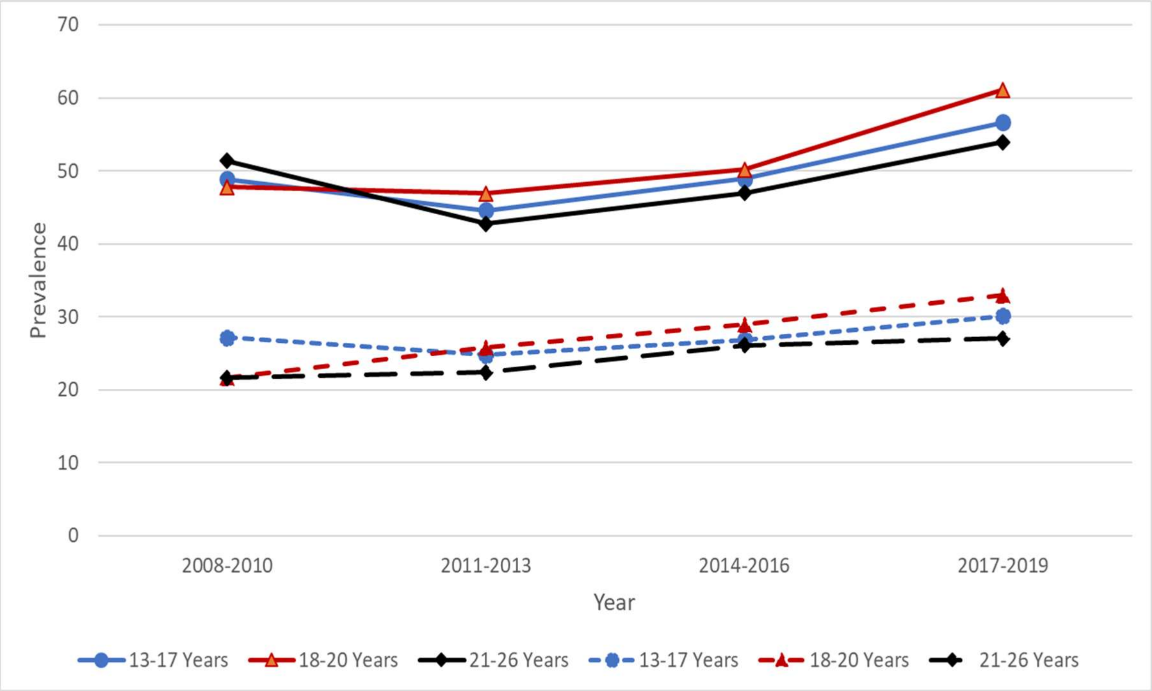

B – Anal HPV

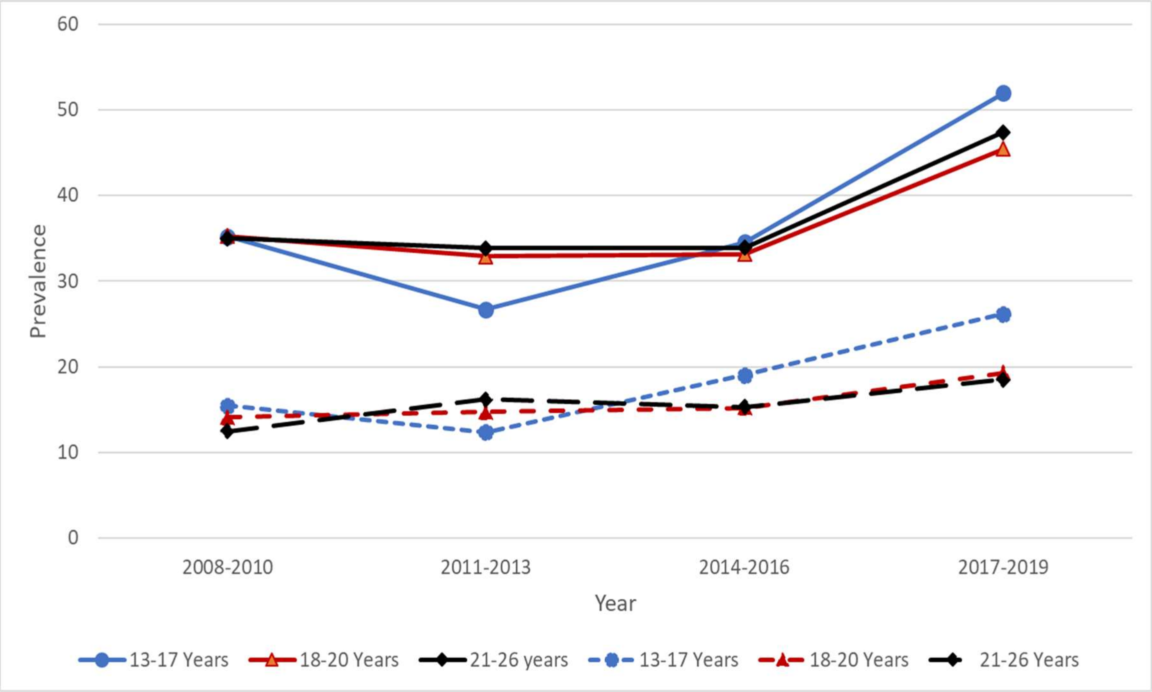

eFigure 3. Prevalence of Cervical and Anal HPV by year and age category.

The baseline prevalence (i.e., at study entry) of Cervical (Panel A) and Anal (Panel B) HPV all types (solid lines), and non-vaccine high-risk HPV types (dashed lines) over time are shown. Prevalence for each time period are stratified by age separated into three age categories - 13-17 years (blue lines with circles), 18-20 years (red lines with triangles) and 21-26 years (black lines with diamonds).

© 2021 Schlecht NF et al. *JAMA Network Open.*

A - Cervical HPV all types tested

B – Cervical non-vaccine high-risk HPV

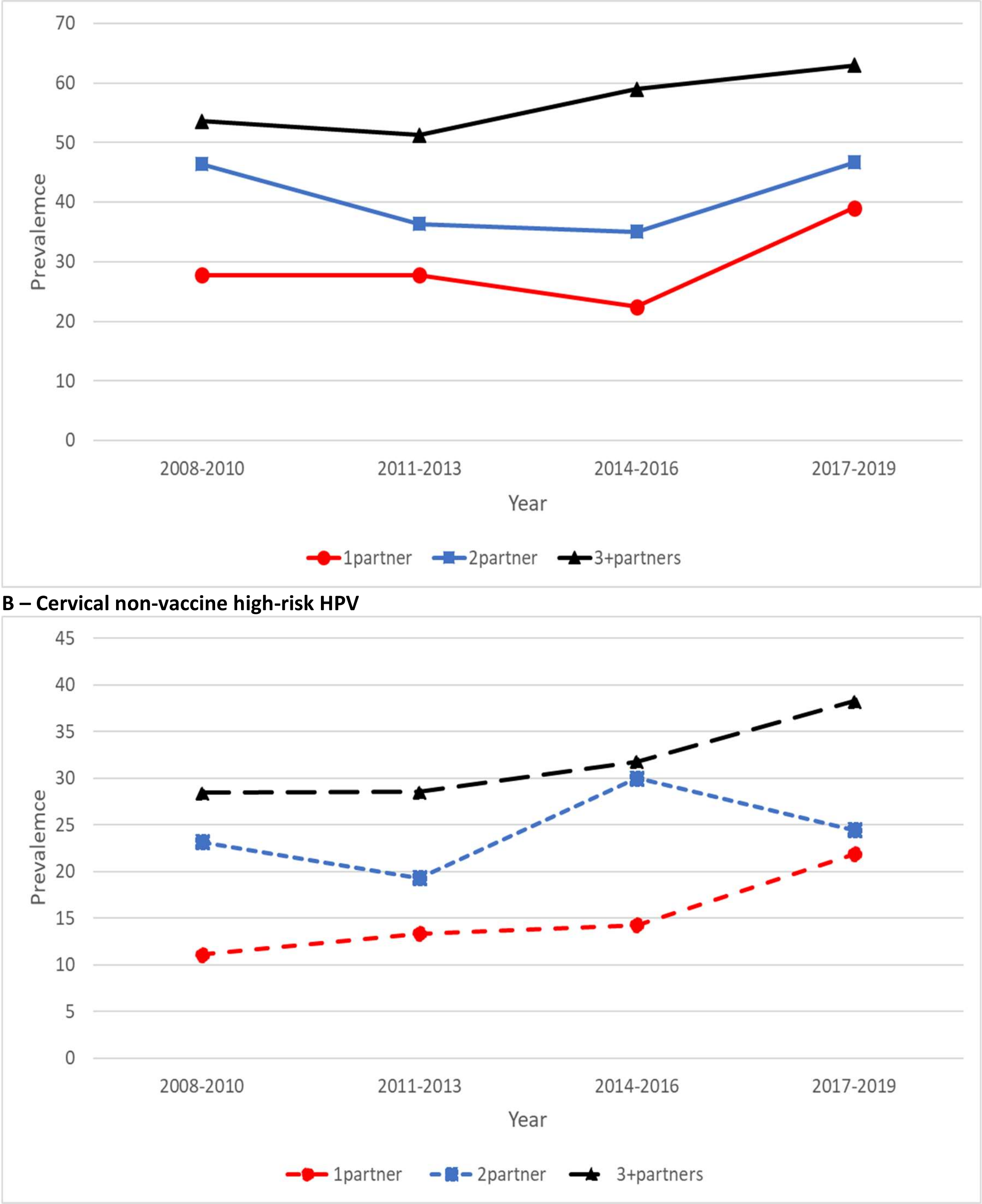

eFigure 4. Prevalence of Cervical HPV by year and number of partners

The baseline prevalence (i.e., at study entry) of cervical HPV all types (Panel A) and non-vaccine high-risk HPV types (Panel B) over time are shown stratified by number of sexual partners in the past 6 months separated into three categories - 1 partner (red lines with circles), 2 partners (blue lines with squares), and 3 or more partners (black lines with triangles).

© 2021 Schlecht NF et al. *JAMA Network Open.*

eTable. Adjusted relative odds of detection of Cervical and Anal HPV over time			
	Vaccine HPV types	Non-vaccine high-risk HPV	All HPV types combined
**Prevalent + Incident Cervical HPV detection**			
**Calendar year at entry (years since 2008)**	**0.81 (0.67, 0.98)**	**1.08 (1.04, 1.13)**	**1.06 (1.02, 1.10)**
Age at visit (years since 13)	1.01 (0.90, 1.13)	1.02 (0.96, 1.08)	1.03 (0.99, 1.08)
Age at coitarche (years)	0.82 (0.64, 1.06)	0.99 (0.91, 1.08)	0.98 (0.91, 1.06)
# vaccine doses received (3 vs. 1-2)	0.53 (0.19, 1.45)	-	1.31 (0.91, 1.89)
Age at first vaccine dose (years)	1.04 (0.75, 1.45)	-	-
# of sexual in past 6 months (2 vs. <2)	0.94 (0.55, 1.62)	1.26 (1.02, 1.55)	1.51 (1.26, 1.80)
# of sexual in past 6 months (3+ vs. <2)	0.86 (0.47, 1.58)	1.55 (1.24, 1.95)	1.86 (1.47, 2.35)
Condom use during recent sex (always vs. not)	-	1.20 (0.93, 1.53)	1.23 (1.00, 1.52)
History of *Chlamydia* (yes vs. no)	-	1.44 (1.17, 1.79)	1.58 (1.30, 1.93)
Cervical co-infection with a vaccine type (yes vs. no)	-	2.38 (1.61, 3.53)	-
**Incident Cervical HPV**			
**Calendar year at entry (years since 2008)**	**0.92 (0.81,1.05)**	**1.04 (0.98,1.10)**	**1.04 (0.98,1.11)**
Age at visit (years since 13)	1.14 (0.99,1.31)	1.04 (0.97,1.12)	1.10 (1.04,1.17)
Age at coitarche (years)	0.77 (0.58,1.01)	1.04 (0.92,1.17)	0.97 (0.87,1.09)
# vaccine doses received (3 vs. 1-2)	1.10 (0.26,4.66)	-	1.27 (0.73,2.20)
Age at first vaccine dose (years)	1.02 (0.77,1.37)	-	-
# of sexual in past 6 months (2 vs. <2)	0.83 (0.40,1.73)	1.33 (1.01,1.75)	1.62 (1.24,2.12)
# of sexual in past 6 months (3+ vs. <2)	1.03 (0.47,2.22)	1.64 (1.23,2.19)	1.92 (1.26,2.91)
Condom use during recent sex (always vs. not)	-	1.13 (0.83,1.54)	1.25 (0.96,1.64)
History of *Chlamydia* (yes vs. no)	-	1.34 (1.02,1.76)	1.64 (1.21,2.22)
Cervical co-infection with a vaccine type (yes vs. no)	-	1.56 (0.94,2.60)	-
**Prevalent + Incident Anal HPV detection**			
**Calendar year at entry (years since 2008)**	**0.88 (0.73, 1.06)**	**1.11 (1.05, 1.17)**	**1.10 (1.05, 1.14)**
Age at visit (years since 13)	1.12 (1.01, 1.25)	1.05 (0.99, 1.11)	1.08 (1.04, 1.13)
Age at coitarche (years)	0.82 (0.61, 1.11)	0.96 (0.87, 1.05)	0.95 (0.89, 1.02)
# vaccine doses received (3 vs. 1-2)	0.58 (0.19, 1.83)	-	0.74 (0.50, 1.11)
Age at first vaccine dose (years)	1.17 (0.84, 1.61)	-	-
Anal sex in past 6 months (yes vs. no)	0.81 (0.37, 1.78)	1.29 (1.00, 1.65)	1.26 (1.02, 1.57)
History of *Chlamydia* (yes vs. no)	-	1.52 (1.20, 1.93)	1.38 (1.13, 1.68)
Anal co-infection with a vaccine type (yes vs. no)	-	2.46 (1.40, 4.32)	-
**Incident Anal HPV**			
**Calendar year at entry (years since 2008)**	**1.02 (0.86,1.21)**	**1.10 (1.03,1.17)**	**1.12 (1.06,1.18)**
Age at visit (years since 13)	1.15 (1.01,1.31)	1.07 (1.00,1.14)	1.12 (1.06,1.18)
Age at coitarche (years)	0.66 (0.45,0.95)	0.97 (0.86,1.09)	0.94 (0.85,1.03)
# vaccine doses received (3 vs. 1-2)	1.30 (0.19,8.85)	0.79 (0.41,1.50)	0.74 (0.42,1.31)
Age at first vaccine dose (years)	1.51 (1.07,2.13)	-	-

© 2021 Schlecht NF et al. *JAMA Network Open.*

Anal sex in past 6 months (yes vs. no)	0.74 (0.30,1.81)		1.34 (1.00,1.80)		1.12 (0.83,1.52)	
History of *Chlamydia* (yes vs. no)	-		-		1.23 (0.95,1.61)	
Anal co-infection with a vaccine type (yes vs. no)	-		3.08 (1.69,5.60)		-	

Odds ratios (and 95% confidence intervals) were estimated by multivariable logistic regression using generalized estimating equations (GEE) adjusting for intra-subject correlations between repeat visit measures, assuming exchangeable correlation based on quasi-likelihood information criterion with robust standard errors, and mutually adjusting for covariates listed in each model. Incident infections were restricted to participants who tested negative at baseline for vaccine types, non-vaccine high-risk HPV types and all tested HPV types, respectively.

© 2021 Schlecht NF et al. *JAMA Network Open.*
